# Revitalizing maize downy mildew management: harnessing new-generation fungicides and host plant resistance

**DOI:** 10.1186/s12870-024-05882-z

**Published:** 2025-02-17

**Authors:** G. Jadesha, M. S. Kitturmath, P. Mahadevu, Chikkappa G. Karjagi, Zahoor Ahmed Dar, H. C. Lohithaswa, D. Deepak

**Affiliations:** 1 Zonal Agricultural Research Station, V.C. Farm, University of Agricultural Sciences, Bangalore, Karnataka India; 2 ICAR-Indian Institute of Maize Research, Unit Office, PUSA Campus, New Delhi, India; 3 Dryland Agriculture Research Station, Srinagar, Jammu and Kashmir India; 4 Zonal Agricultural Research Station, GKVK, University of Agricultural Sciences, Bangalore, Karnataka India; 5 Department of Mechatronics, Manipal Institute of Technology, Manipal Academy of Higher Education (MAHE), Manipal, Karnataka India

**Keywords:** Maize downy mildew, Survey, Fungicides, Host plant resistance, LCMS, Phenolics and flavonoids

## Abstract

**Background:**

Maize Downy Mildew (MDM) is a devastating disease in the humid sub-tropical/tropical regions of Asia. In this study, the prevalence of MDM during the rainy *Kharif* seasons of south Karnataka state (India) ranged between 6.8% (2018) and 19.1% (2022). The research evaluated new fungicidal treatments and assessed the genetic tolerance of maize lines to develop robust management strategies that enhance maize productivity and stability.

**Results:**

During the *Kharif* seasons of 2021 and 2022, we conducted field trials to evaluate the effectiveness of six different fungicides, both individually and in combination. The most effective approach combined seed treatment with Metalaxyl (4%) and Mancozeb (64%) WP, followed by a foliar spray of Azoxystrobin (18.2%) and Difenoconazole (11.4%) SC. This treatment reduced MDM incidence by 97.6% and increased maize yield up to 85.6 quintals per hectare, with a benefit-cost ratio of 2.2. Additionally, screening of 317 maize inbred lines in *Kharif* 2019 identified 22 lines with stable MDM resistance over nine consecutive *Rabi* and *Kharif* seasons, indicating their potential for sustained resistance. Liquid Chromatography-Mass Spectrometry (LC-MS) analysis revealed significant increases in eighteen phenolic compounds and fifteen flavonoid compounds in resistant maize genotypes. Specifically, resistant genotypes exhibited elevated levels of salicylic acid (4.2 to 9.2-fold), p-Coumaric acid (3.7 to 4.8-fold), o-Coumaric acid (4.5 to 7.4-fold), Caffeic acid (2.4 to 3.1-fold), and Ferulic acid (2.3 to 2.8-fold). Flavonoid levels also increased, with Naringenin ranging from 34.4 µg/g in African Tall to 130 µg/g in MAI 224, Catechin from 22.9 µg/g in African Tall to 124.4 µg/g in MAI 10, and Epicatechin from 1.3 µg/g in African Tall to 8.2 µg/g in MAI 10. These heightened levels contribute to a robust chemical defence mechanism against *Peronosclerospora sorghi*.

**Conclusions:**

This study provides crucial insights into managing MDM through host plant resistance and fungicidal treatments. We identified 22 resistant inbred lines as valuable genetic resources for breeding MDM-resistant maize hybrids. Enhanced levels of specific phenolic and flavonoid compounds in these resistant genotypes suggest a robust chemical defence mechanism, essential for developing resilient crops. Our findings offer practical recommendations for improving maize production and ensuring crop security in MDM-affected regions. Integrating these resistant maize lines and effective fungicidal treatments can significantly advance sustainable agricultural practices, contributing to crop resilience and food security in areas prone to MDM.

**Supplementary Information:**

The online version contains supplementary material available at 10.1186/s12870-024-05882-z.

## Introduction

Maize (*Zea mays* L.), usually known as corn, holds a vital position as a staple food and an important agricultural commodity both on a global scale and within India. With its remarkable resourcefulness covering from food to feed, fuel, and various industrial applications, maize stands as the foremost cereal crop in terms of production worldwide [1, 2]. In Indian context, maize ranks third among the essential food grains, following wheat and rice, playing a vital role in agricultural practices, economic contribution, and employment generation [3, 4].

Maize plays a crucial role in combating malnutrition and offers significant economic benefits to marginalized farming communities in India. Its cultivation can improve food security and provide a stable income, helping uplift these communities [3]. Conservation agriculture offers promising prospects for augmenting maize yields and ensuring food security, particularly in the face of climate change challenges as highlighted in [5], adopting these practices can significantly enhance agricultural productivity. The recognition of maize’s capacity to enhance farmers’ incomes resonates with the overarching goal of doubling agricultural earnings in India, with Karnataka emerging as a frontrunner, accounting 17% to the nation’s maize production [6]. Although many achievements have been made in the past years, maize industry is limited and has ample opportunity for growth that can be leveraged through better agricultural practices to create a growth trajectory sustained over time. In 2021, maize was produced globally up to 1.21 billion tonnes in which India produced 31.65 million tonnes [6].

Plant diseases and insect pests remain persistent challenges to maize production, impacting approximately 50% of maize-growing areas in Asia. These issues can lead to potential yield losses of up to 14% [7]. Among these challenges, downy mildew caused by *Peronosclerospora sorghi* has emerged as a significant factor since the early 1900s, posing a substantial threat to global food security, with yield losses of up to 30% in affected regions [8]. Despite the introduction of resistant cultivars and the use of metalaxyl fungicides, severe downy mildew incidence persists in localized areas [9], necessitating the revitalization of sustainable strategies to safeguard maize productivity. Extensive research has focused on the efficacy of systemic fungicides in mitigating downy mildew, showcasing notable success through seed treatments and foliar sprays [10]. However, despite the potential for significant yield increases with these treatments [11, 12], adoption remains low in certain regions due to knowledge gaps among farmers [13, 14]. Nevertheless, the increasing demand for maize across Asia suggests a proactive approach is underway. In the context of traditional management relying solely on metalaxyl, to which the pathogen developed resistance [15], the imperative to adopt new-generation fungicides becomes evident. Research has yielded diverse products with novel modes of action, significantly impacting disease control. These innovative fungicides address resistance management, regulatory challenges, and customer expectations, with unique modes of action playing a pivotal role. Novel compounds like phenylpyrroles, anilinopyrimidines, and strobilurin analogues have been introduced in recent years, impacting vital cellular processes [16]. The increasing demand for maize across Asia suggests a proactive approach is underway.

Despite advancements in maize cultivation and management practices, MDM remains a severe threat, causing substantial yield losses and affecting productivity. Current management practices, including fungicide use and genetic resistance, have not fully addressed the issue, leaving gaps in effective control. This study seeks to fill this gap by evaluating new fungicidal treatments and identifying maize inbred lines with stable resistance to MDM, while also investigating the biochemical mechanisms, specifically phenolic and flavonoid compounds, that contribute to this resistance.

Host plant resistance represents a cost-effective and efficient approach for controlling downy mildew disease in maize [17–19]. Resistant varieties have been effectively deployed across the Americas and Asia, addressing the challenges posed by this destructive disease [20, 21]. However, despite these efforts, the severe incidence of downy mildew persists in localized areas [9].Upon pathogen infection, plants activate a diverse array of defence responses, including the synthesis of new proteins that directly or indirectly influence the pathogenesis course [22, 23]. These induced proteins encompass cell wall proteins, enzymes involved in phenols and flavonoid metabolism, toxic proteins, antimicrobial proteins (enzyme inhibitors), oxidative enzymes, lytic enzymes, and a diverse group collectively known as pathogenesis-related proteins [24]. The early release of preformed phenolicacids and their subsequent intensified production as a response to the stimulation of phenylpropanoid metabolism are integral components of plant resistance reactions to diseases [25]. The phenylpropanoid pathway, crucial for producing secondary metabolites like lignin, phenolic acids, and phytoalexins, enhances cell wall mechanical rigidity and strength while forming barriers to pathogen infection [26]. Previous studies have emphasized the correlation in increased host resistance with high phenolic compound content [27, 28], highlighting the significance of biochemical understanding in host–pathogen interactions. The diversity of downy mildew pathogens, such as *Peronosclerospora sorghi* prevalent in peninsular India [29] and those causing significant economic losses in Asia [30], underscores the importance of developing comprehensive strategies to fortify maize against this formidable threat. A comprehensive understanding of host–pathogen interactions at the biochemical level is imperative.The objective of this study was to identify stable resistant inbreds against MDM disease and to explore the associated biochemical intricacies. Additionally, the study aimed to compare the levels of phenolic and flavonoid compounds in maize genotypes resistant and susceptible to MDM.

Our study adopts a comprehensive approach, encompassing field surveys, experiments involving innovative fungicide-based management, identification, and validation of resistant inbreds. We explore the intricate dynamics between susceptible and resistant lines, focusing especially on phenolic and flavonoid responses, as well as maize resistance against downy mildew disease. Through these endeavours, our research aims to make significant contributions to the understanding of this critical domain. The subsequent sections delve into the details of our methodologies, findings, and the implications that arise from the multifaceted insights gained.

## Materials and methods

### Assessment of MDM occurrence and distribution

A roving survey was conducted to assess the occurrence and distribution of MDM disease in 19 taluks across seven districts (Supplementary Fig. 1) in southern Karnataka during the rainy seasons of the years 2018, 2019, 2021, and 2022. Within each taluk, fields were randomly selected for inclusion in the survey. In each selected field, five plots measuring 10 m × 10 m were demarcated and distributed randomly throughout the field. Observations on MDM were made within each plot for MDM incidence and the per cent disease incidence (PDI) for each plot was recorded. The PDI was calculated for each plot by dividing the number of plants infected plants by the total number of plants and multiplying the result by 100. To determine the average incidence of MDM in each field, the PDI values from all five plots within the field were averaged. This average PDI represented the overall disease incidence within that particular field. The average PDI of each field was then used to calculate the average PDI for each specific taluk. This was achieved by summing the average PDI values from all the fields surveyed within that particular location and dividing it by the total number of fields surveyed. The collected data allowed comprehensive analysis of the occurrence and distribution of MDM across the surveyed taluks and districts. This information will contribute to a deeper understanding of MDM dynamics and support informed decision-making for effective disease management strategies.

### Unleashing the potential of fungicide treatments

The experiments were conducted during the *Kharif* seasons of 2021 and 2022 on downy mildew-affected soil maintained in maize research plots at the Zonal Agricultural Research Station, V.C. Farm (latitude 12.56º, longitude 76.82º). For the study, we selected a susceptible maize hybrid, MAH 14 − 5. Six fungicides, each with label claims for maize crops and downy mildew disease, were tested [31]. Details regarding the fungicides, their dosages, timing, and methods of application are provided in Table 1. The experimental plots were each 5 m in row length, with 7 rows per plot, totalling 21 square meters. Plant density was 0.6 m between rows and 0.2 m between plants. The experimental design used was a Randomized Complete Block Design (RCBD). Artificial inoculation was carried out at the two-leaf stage using the method described by [32]. Foliar sprays were applied once at 30–50 days after sowing, depending on disease occurrence. The inoculation process began when the seedlings reached the two-leaf stage and continued until the plants were 15 days old and showing systemic symptoms. The inoculum concentration used was 10^− 5^ zoospores per ml of water. The downy mildew strain employed in the study was selected from our national downy mildew screening centre, where the pathogen is maintained on maize plants throughout the year to ensure a consistent and reliable strain for our experiments. The percentage of disease incidence was noted at 30 days after the plantings using the formula: (number of infected plants / total number of plants) multiplied by 100. This technique was described by [33].

**Table 1:** Details of fungicides used in the study

S. No.	Fungicides^#^	Dosage (Per kg seeds or Per liter of water)	Application^*^
T-1	Mancozeb 75% WP	2.0–2.5 g	Seed Dressing (SD)
T-2	Metalaxyl 35% WS	2.4 g	Seed Dressing (SD)
T-3	Metalaxyl − 4% and Mancozeb − 64% (68% WP)	3.0 g	Seed Dressing (SD)
T-4	Carbendazim 12%+ Mancozeb 63% WP	2.0 g	Foliar Spray (FS)
T-5	Azoxystrobin 18.2% w/w + Difenoconazole 11.4% w/w SC	1.0 ml	Foliar Spray (FS)
T-6	Azoxystrobin 18.2% w/w + Cyproconazole 7.3% w/w SC	1.0 ml	Foliar Spray (FS)
T-7	T3 + Carbendazim 12%+ Mancozeb 63% WP	3.0 g + 2.0 g	SD + FS
T-8	T3 + Azoxystrobin 18.2% w/w + Difenoconazole 11.4% w/w SC	3.0 g + 1.0 ml	SD + FS
T-9	T3 + Azoxystrobin 18.2% w/w + Cyproconazole 7.3% w/w SC	3.0 g + 1.0 ml	SD + FS
T-10	Inoculated Control (Without any fungicide application)	-	-

# all the fungicides are having the label claim for crop and disease. * Single Foliar Spray done at 30–50 days after sowing based on occurrence of disease

### Plant material and seed sources for MDM resistance

The assessment of maize inbreds for resistance to MDM was conducted following the protocols outlined by [32], utilizing the spreader row technique. During Kharif 2019, a total of 317 unique maize inbreds were screened. The plant materials used in this study included maize inbred lines and composites sourced from maize research institutes and breeding programs. Specifically, Mandya Inbred Lines (MAI), Pathology Trial Inbred Lines (PT), Nagenahalli Inbred Lines (NAI), Popcorn Lines (POP-1, PC), Fusarium Stalk Rot Resistant Lines (FSR), and Sweet Corn Lines (SC, SC-P) were obtained from the All India Coordinated Research Project (AICRP), MAIZE, Mandya, Karnataka. Haryana-Karnal Inbred Lines (HKI) were sourced from Haryana Agricultural University (HAU), Hisar. Quality Protein Maize (QPM) was provided by the Indian Institute of Maize Research (IIMR), Winter Nursery Center, Hyderabad, Telangana. Lines from the International Maize and Wheat Improvement Center (CIMMYT), Hyderabad, Telangana, included CIMMYT Advanced Lines (CAL), CIMMYT Maize Lines (CML), SKV Lines (developed by Surinder Kumar Vasal), Sudha Nair Lines (SN; developed by Dr. Sudha Nair), and Z Lines (developed by Dr. Zaidi). Additionally, Vivekananda Inbred Lines (V) were developed by Vivekananda Parvatiya Krishi Anusandhan Sansthan (VPKAS), ICAR. Detailed genotype information, including pedigree data and breeding history, is provided in Supplementary Tables 2 and 3.

The experiment was laid out in a randomized complete block design (RCBD) with two replications. Each replication consisted of two rows, with a row length of 3 m. The spacing was maintained at 0.2 m between plants and 0.6 m between rows. Disease incidence was recorded 30 days after sowing. Based on the Percentage Disease Incidence (PDI), the genotypes were classified into four categories: resistant (PDI 0 to 10%), moderately resistant (PDI 10.1 to 25.0%), moderately susceptible (PDI 25.1 to 50.0%), and susceptible (PDI greater than 50.0%) [32].

### Validation of resistance in Maize inbreds against MDM: a multi-season analysis

The validation of resistance in maize inbreds was conducted over multiple seasons: Rabi 2019-20, Kharif 2020, Rabi 2020-21, Kharif 2021, Rabi 2021-22, Kharif 2022, Rabi 2022-23, Kharif 2023, and Rabi 2023-24. A total of 22 resistant genotypes, as detailed in Table 2, were selected for this study. The experiment was arranged in a randomized complete block design (RCBD) with three replications. Each replication consisted of two rows, each with a length of 3 m. The row-to-row spacing was maintained at 0.6 m, and the plant-to-plant spacing was 0.2 m.

**Table 2:** Validation of resistance in Maize Inbreds against *Peronosclerospora sorghi*: a multi-season analysis

S. No.	Inbred	Per cent Disease Incidence*											
		^#^K- 2019	^#^*R*- 2019-20	^#^K- 2020	^#^*R*- 2020-21	^#^K- 2021	^#^*R*- 2021-22	^#^K- 2022	^#^*R*- 2022-23	^#^K- 2023	^#^*R*- 2023-24	Mean PDI	Disease Reaction
1	MAI 1	0.0	0.0	0.0	0.0	0.0	0.0	0.0	0.0	0.0	0.0	**0.0**	**R**
2	MAI 10	0.0	0.0	0.0	0.0	0.0	0.0	0.0	0.0	0.0	0.0	**0.0**	**R**
3	MAI 20	0.0	0.0	0.0	0.0	0.0	0.0	0.0	0.0	0.0	0.0	**0.0**	**R**
4	SKV 50	0.0	0.0	0.0	0.0	0.0	0.0	0.0	0.0	0.0	0.0	**0.0**	**R**
5	MAI 142	0.0	0.0	0.0	0.0	0.0	0.0	0.0	0.0	0.0	0.0	**0.0**	**R**
6	MAI 723	0.0	0.0	0.0	0.0	0.0	0.0	0.0	0.0	0.0	0.0	**0.0**	**R**
7	Z 485 − 20	0.0	0.0	0.0	0.0	0.0	0.0	0.0	0.0	0.0	0.0	**0.0**	**R**
8	MAI 224	0.0	0.0	0.0	0.0	0.0	0.0	0.0	0.0	0.0	0.0	**0.0**	**R**
9	MAI 753	0.0	0.0	0.0	0.0	0.0	0.0	0.0	0.0	0.0	0.0	**0.0**	**R**
10	QPM 28	0.0	0.0	0.0	0.0	0.0	0.0	0.0	0.0	0.0	0.0	**0.0**	**R**
11	QPM 26	0.0	0.0	0.0	0.0	0.0	0.0	0.0	0.0	0.0	0.0	**0.0**	**R**
12	1298	0.0	0.0	0.0	0.0	0.0	0.0	0.0	0.0	0.0	0.0	**0.0**	**R**
13	MAI 7	3.6	0.0	0.0	0.0	0.0	0.0	0.0	0.0	0.0	0.0	**0.5**	**R**
14	QPM 25	0.0	0.0	10.0	0.0	5.2	0.0	0.0	0.0	2.5	3.1	**2.1**	**R**
15	NAI 137	0.0	0.0	8.3	0.0	11.0	0.0	0.0	0.0	0.0	0.0	**2.4**	**R**
16	52,067	0.0	0.0	0.0	0.0	0.0	10.0	5.2	4.2	1.1	2.2	**2.4**	**R**
17	CML 451	0.0	0.0	0.0	0.0	0.0	1.5	9.1	5.5	6.2	9.2	**3.2**	**R**
18	MAI 3	3.6	6.2	12.5	6.2	16.2	5.9	7.2	0.0	2.5	0.0	**6.0**	**R**
19	MAI 13	0.0	0.0	0.0	0.0	14.3	7.9	8.5	9.9	16.9	11.1	**6.9**	**R**
20	MAI 12	0.0	0.0	0.0	0.0	16.7	11.5	15.2	10.0	9.5	10.1	**7.3**	**R**
21	MAI 2	4.2	8.3	13.3	8.3	16.7	9.5	10.5	5.5	0.0	0.0	**7.6**	**R**
22	MAI 16	3.8	0.0	0.0	0.0	20.0	15.5	12.5	16.1	9.5	10.2	**8.8**	**R**
SC	African Tall	95.2	82.5	100.0	88.0	100.0	93.6	91.2	93.5	85.6	93.6	**92.3**	**S**

* Mean of three replications; # K = *Kharif*, # R = *Rabi*; SC = Susceptible Check; R = Resistant; S = Susceptible

Disease incidence was recorded 30 days after sowing (DAS), and the genotypes were classified according to the criteria set by reference [32]. To serve as a control, the susceptible genotype ‘African Tall’ was included in the study. It was sown in two rows and repeated at regular intervals after every five entries to ensure accurate comparison.

### LCMS-based profiling and quantification of phenolic acids and flavonoids in maize leaf extracts

The LCMS analysis of phenolic acids and flavonoids was performed 30 days after sowing, utilizing a technique derived from [34, 35] with some minor modifications. Maize leaf samples were collected, including four stable resistant entries (MAI 10, SKV 50, MAI 723, and MAI 224), selected based on their consistent resistance across multiple seasons in this study. Additionally, one susceptible entry (African Tall) was included for comparative analysis. The maize leaf samples underwent the following steps in the analysis process.

First we mix all the samples’ like known weights in 80% methanol for homogenize them well Further, we centrifuge extracts and then adjusting them to a volume like of 20 mL.

### Evaporation and dilution

The extract will evaporate under vacuum at exactly 45 °C till almost dry; additionally, diluting it with water to a smaller amount like 5 mL.

### Treatment with petroleum ether

Next, treating the already diluted extract about three times with petroleum ether before for collecting as an aqueous layer is necessary.

### Extraction with ethyl acetate

Following that, extract the aqueous layer once more with ethyl acetate using separating funnel twisted.

### Drying and mixing

Once the aqueous layer is removed, the ethyl acetate extract dried under vacuum at room temperature. Consequently, the dry residue is merging with presumably 4 mL of 2 N NaOH and left to hydrolyze overnight.

### Acidification and extraction

Consequently, the mixture is acidified to pH 2 that sounds cool with 5 mL of doubly 2 N HCl before it’s extracted amusingly once more with seemingly 10 mL of ethyl acetate.

### Evaporation and filtration

The ethyl acetate layer containing potentially phenolic acids and flavonoids then wholly evaporated under weird vacuum. The residue pleasingly dissolved in 1 mL of MS-grade methanol and then filtered through kind a 0.2 μm definitely nylon filter before showcasing injecting into LC-MS/MS for somewhat estimating phenolic acids and flavonoids.

## Results

### Spatial and temporal dynamics of Maize Downy Mildew

**Fig. 1:**
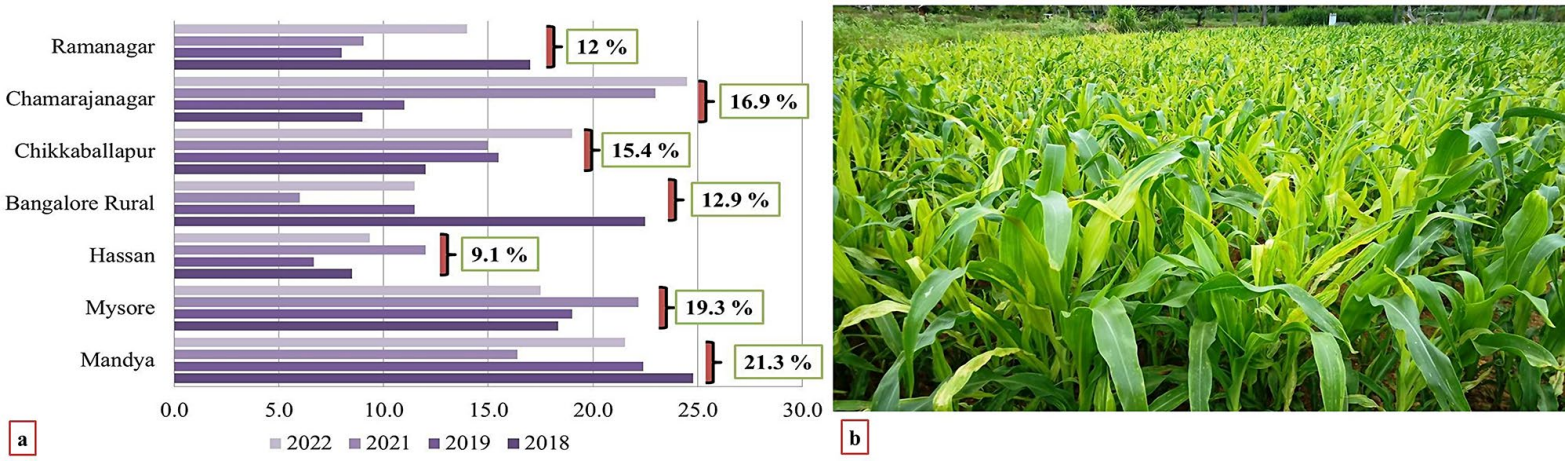
**a)** Exploring spatial and temporal dynamics of Maize Downy Mildew. **b)** MDM Incidence in Farmers’ Fields: 100% Prevalence in 2019 Rainy Season

The incidence of MDM disease as presented in Fig. 1 and Supplementary Table 1.

The spatial analysis revealed significant variations in MDM incidence across surveyed districts. Mandya District showed the highest mean PDI at 21.3%, while Hassan District had the lowest at 9.1%. District wise comparisons ranked from highest to lowest incidence, Mandya (21.3%), Mysore (19.3%), Chamarajnagar (16.9%), Chikkaballapur (15.4%), Bangalore Rural (12.9%), Ramanagar (12.0%), and Hassan (9.1%). These findings provide valuable insights into MDM incidence levels, enhancing comprehension of disease distribution and relative burdens. The temporal dynamics further emphasized the dynamic nature of MDM. Over the span of four years, the incidence of disease showed fluctuations: 2018 (17.1%), 2019 (14.8%), 2021 (15.3%), and 2022 (17.2%).

### Fungicide intervention in controlling maize downy mildew

**Fig. 2:**
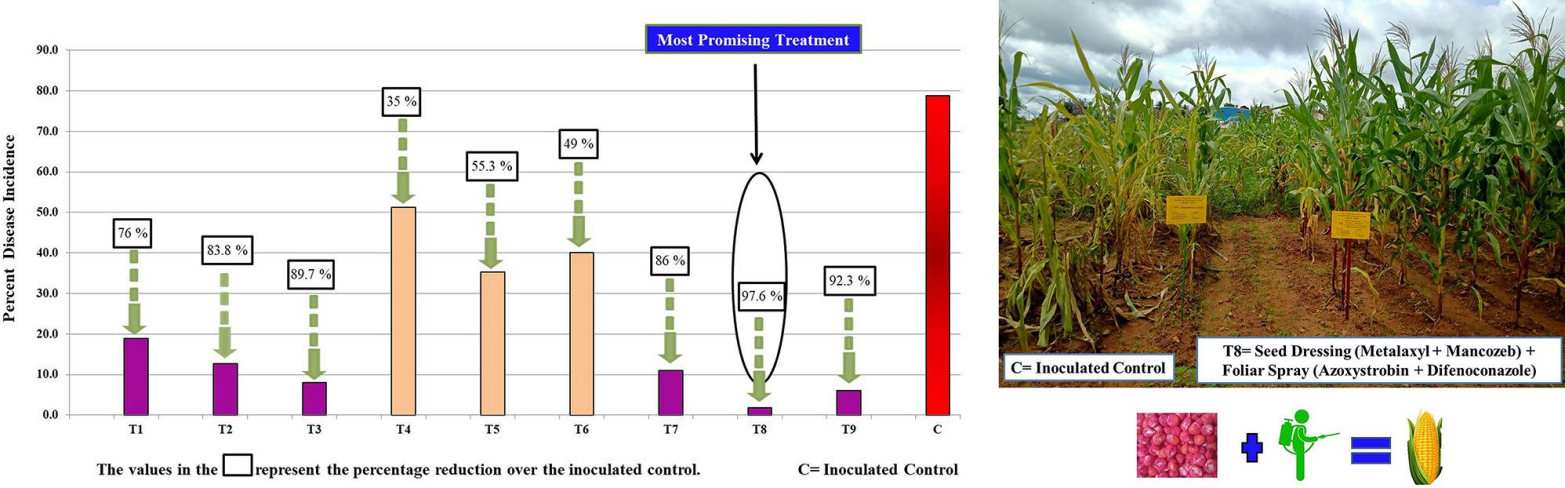
Fungicide interventions in Controlling Maize Downy Mildew

The comparative analysis of fungicide treatments during the *Kharif* seasons of 2021 and 2022 reveals significant variations in their performance (Fig. 2) in terms of PDI and Percent Reduction over Control (PROC). The most effective treatment was T8, demonstrating the lowest PDI of 1.9 and the highest PROC of 97.6 in both seasons, making it the top-performing strategy. Following closely, T3 showed a PDI of 8.1 and a PROC of 89.7, ranking as the second-best management strategy. Additionally, T7 with a PDI of 11.0 and a PROC of 86.0, and T2 with a lower PDI of 12.8 and PROC of 83.8, displayed commendable performances.T1 showed a PDI of 18.9 and a PROC of 76.0. In contrast, the control treatment (T10), without fungicides, recorded the highest PDI value of 78.8, indicating poor crop health and significant disease prevalence. T5, T6, and T4 displayed moderate disease control, with PDIs of 35.2, 40.2, and 51.3, respectively, suggesting limited effectiveness in managing MDM. In a summary, seed treatment followed by foliar spray emerged as the most effective management strategy, while seed treatment alone also exhibits significant efficacy in managing MDM. However, solely relying on foliar spray does not provide sufficient efficacy to manage MDM disease effectively. These findings emphasize the significance of selecting appropriate fungicides and combinations to effectively control diseases, maximize crop productivity during *Kharif* farming, and form a robust integrated disease management strategy.

### Evaluating the yield benefits of fungicide applications for MDM management

**Fig. 3:**
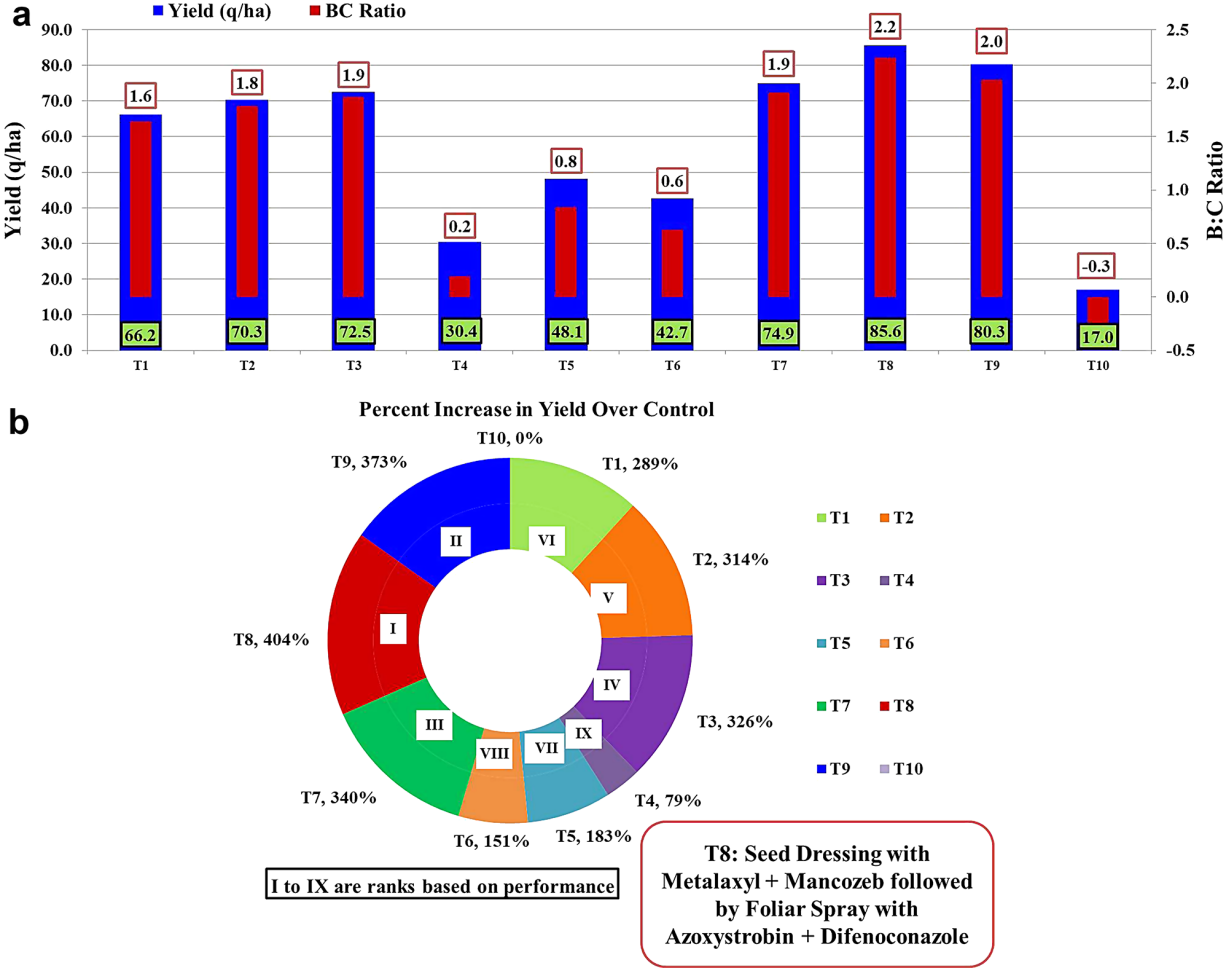
**a** Assessment of Yield and Benefit-Cost Ratio from Fungicide Applications for MDM Management. **b** Per cent Increase in Yield over Control from Fungicide Applications for MDM Management

The complete analysis of fungicide applications during the *Kharif* seasons of 2021 and 2022, as showed in Fig. 3a and b, revealed important differences in their performance about yield, PIOC (Per cent Increase over Control), and B: C ratio (Benefit Cost Ratio).

In terms of yield, the treatments exhibited varying levels of productivity. T8 demonstrated the highest yield at 85.6 q/ha, showcasing its effectiveness in enhancing crop growth and maximizing output besides its best efficacy in managing the MDM disease. Subsequently, T9 followed closely with a yield of 80.3 q/ha, and T7 with 74.9 q/ha. T3 recorded a yield of 72.5 q/ha, and T2 with 70.3 q/ha. Considering PIOC, which represents the percentage increase over the control in yield, T8 emerged as the most effective treatment, followed by T9, T7, T3, and T2. These treatments displayed significant increases in yield compared to the control. In terms of the BC ratio, reflecting economic viability, T8 exhibited the highest ratio at 2.2, indicating a positive return on investment and a favourable cost-benefit balance. It was closely followed by T9 (2.0), T7 (1.9), and T3 (1.9). Notably, T10 (Control) served as the inoculated control, recording the lowest yield at 6.8 q/acre and a negative BC ratio of -0.3. These results underscore the importance of implementing effective fungicide treatments to combat plant diseases and safeguard crop productivity. In summary, T8 seed treatment with Metalaxyl − 4% and Mancozeb − 64% at 3.0 g per kg of seeds followed by foliar spray of Azoxystrobin 18.2% w/w + Difenoconazole 11.4% w/w SC at 1 ml per litre of water displayed the most favourable results in terms of MDM management, yield, and BC ratio.

### Maize inbreds and their susceptibility to maize downy mildew

A total of 317 maize inbreds underwent evaluation for their susceptibility to MDM (Supplementary Table 4). Disease incidence was gauged using the Percentage Disease Incidence (PDI). Among these inbreds, 32 displayed a PDI of less than 10%, categorizing them as Resistant (R). Notably, among these 32 resistant inbreds, 27 recorded no incidence, including 1298, 1,926,015, CML 451, MAI 1, MAI 12, MAI 13, MAI 142, MAI 20, MAI 224, MAI 723, MAI 753, MAI-10, MAI-105, MAI-163, MAI-191, MAI-2-1, MAI-729, MIA 7, NAI 137, NAI-175, QPM 25, QPM 26, QPM 28, SKV 50, SN-485-152, V-79, and Z 485 − 20. Additionally, 18 inbreds were classified as Moderately Resistant, with PDIs ranging from 10.1 to 25.0%. In contrast, 61 inbreds exhibited a Moderately Susceptible reaction, with PDIs ranging from 25.1 to 50.0%. Moreover, 206 inbreds were identified as susceptible to MDM disease, with PDIs exceeding 50%. Among these, 149 inbreds displayed a 100% incidence rate. Notably, the Susceptible check African Tall exhibited a PDI of 95.2, indicating its high susceptibility to MDM disease. These results underscore the varied responses of maize inbreds, spanning from zero to 100% MDM incidence.

### Evaluation of maize inbreds’ stability against MDM: a multi-season analysis

A comprehensive evaluation was conducted to validate resistance in maize inbreds against MDM, spanning multiple growing seasons (Table 2). The PDI was utilized to scale disease reaction. Among the 22 resistant inbreds, 12 inbreds demonstrated consistent resistance with a PDI of 0.0 across all seasons. Notable inbreds in this category include MAI 1, MAI 10, MAI 20, SKV 50, MAI 142, MAI 723, Z 485 − 20, MAI 224, MAI 753, QPM 28, QPM 26, and 1298. Additionally, other inbreds displayed varying levels of resistance, with PDIs ranging from 0.5 to 8.8, such as MAI 7, QPM 25, NAI 137, 52,067, CML 451, MAI 3, MAI13, MAI12, MAI2 and MAI 16. However, susceptible check African Tall exhibited high susceptibility consistently across all seasons, with PDIs ranging from 82.5 to 100.0%. This comprehensive assessment underscores the stability of resistance in certain maize inbreds against MDM, with African Tall being notably susceptible throughout the study period.

### Phenolic profiling reveals Maize genotypic resistance to maize downy mildew

The phenolic acid content (µg/g) analysis (Table 3.) among the four resistant genotypes (MAI 10, SKV 50, MAI 723, and MAI 224) and the susceptible African Tall highlighted distinct concentrations, shedding light on their potential role in resistance to MDM. Salicylic acid, a crucial signalling compound in plant defence, showed variations across entries. MAI 723 exhibited the highest concentration at 28.6 µg/g, emphasizing its potential role in resistance. In contrast, the susceptible African Tall recorded the lowest concentration at 3.1 µg/g. P-coumaric acid, recognized for its antioxidant properties and contribution to disease resistance, displayed significant variability. MAI 723 showcased the highest content (259.4 µg/g), underlining its potential role in resistance. Conversely, African Tall had the lowest concentration (92.1 µg/g).O-coumaric acid, another phenolic compound associated with plant stress responses, demonstrated noteworthy concentration differences. SKV 50 displayed the highest content (355.9 µg/g), while African Tall exhibited the lowest (86.9 µg/g).Similarly, in MAI 723 Caffeic acid and Ferulic acid exhibited highest in 283.9 µg/g and 116.8 µg/g respectively when compare to susceptible African tall. Other phenolic acids, including vanillic acid, gallic acid benzoic acid, p-hydroxy benzoic acid, 3-hydroxy benzoic acid, t-cinnamic acid, 2,4-dihydroxybenzoic acid, gentisic acid, protocatechuic acid, syringic acid, sinapic acid, ellagic acid, and chlorogenic acid exhibited diverse but little concentrations across genotypes. This comprehensive analysis enhances our understanding of the biochemical responses in resistant and susceptible genotypes of maize MDM.

**Table 3:** LCMS Analysis of Phenolic Acid Content in resistant maize genotypes vs. susceptible African Tall

Phenolic acids (µg/g)*	African Tall	MAI 10	SKV 50	MAI 723	MAI 224	SEM	CD 0.01	CV
o-Coumaric acid	86.9 ^**e**^	217.3 ^**d**^	355.9 ^**a**^	292.3 ^**b**^	273 ^**c**^	0.09	0.39	0.07
p-Coumaric acid	92.1 ^**e**^	239.3 ^**b**^	202.9 ^**d**^	259.4^**a**^	230.9 ^**c**^	0.09	0.28	0.06
Caffeic acid	52.5 ^**e**^	240.5 ^**b**^	231.6 ^**c**^	283.9 ^**a**^	223.5 ^**d**^	0.06	0.25	0.05
Ferulic acid	41.5 ^**e**^	112.2 ^**b**^	96.2 ^**c**^	116.8 ^**a**^	95.5 ^**d**^	0.09	0.26	0.12
Salicylic acid	3.1 ^**e**^	13.1 ^**d**^	25.3 ^**b**^	28.6 ^**a**^	15.4 ^**c**^	0.03	0.12	0.30
Gallic acid	5.6 ^**c**^	13.4 ^**a**^	4.1 ^**d**^	3.3 ^**e**^	12.5 ^**b**^	0.03	0.13	0.67
Vanillic acid	5.0 ^**d**^	5.2 ^**c**^	3.8 ^**e**^	6.2 ^**a**^	5.7 ^**b**^	0.03	0.13	1.00
Benzoic acid	1.2 ^**b**^	1.1 ^**c**^	1.1 ^**c**^	1.1 ^**c**^	1.5 ^**a**^	0.02	0.09	3.14
t-Cinnamic acid	1.7 ^**a**^	1.1 ^**b**^	1.1 ^**b**^	1.0 ^**b**^	1.2 ^**b**^	0.07	0.3	10.74
Gentisic acid	1.6 ^**a**^	1.1 ^**b**^	1.0 ^**b**^	1.5 ^**a**^	1.2 ^**b**^	0.07	0.28	9.42
Protocatechuic acid	0.4 ^**c**^	0.5 ^**b**^	0.3 ^**d**^	0.3 ^**d**^	0.6 ^**a**^	0.02	0.09	8.42
2,4-dihydroxybenzoic acid	0.2 ^**c**^	0.5 ^**ab**^	0.6 ^**a**^	0.5 ^**ab**^	0.4 ^**b**^	0.04	0.18	15.12
p-hydroxy benzoic acid	0.2 ^**a**^	0.1 ^**b**^	0.1 ^**b**^	0.1 ^**b**^	0.1 ^**b**^	0.01	0.05	19.14
3-Hydroxy benzoic acid	0.1 ^**a**^	0.1 ^**a**^	0.0 ^**b**^	0.1 ^**a**^	0.1 ^**a**^	0.01	0.05	19.14
Sinapic acid	0.2 ^**b**^	0.1 ^**c**^	0.5 ^**a**^	0.2 ^**b**^	0.1 ^**c**^	0.03	0.09	16.07
Syringic acid	0 ^**a**^	0.1 ^**a**^	0 ^**a**^	0 ^**a**^	0 ^**a**^	0.01	0.02	43.74
Ellagic acid	0 ^**a**^	0 ^**a**^	0 ^**a**^	0.1 ^**a**^	0 ^**a**^	0.01	0.02	19.64
Chlorogenic acid	0 ^**a**^	0 ^**a**^	0 ^**a**^	0 ^**a**^	0 ^**a**^	0	0	0.00

* Mean of four replications, SEM = Standard Error of the Mean, CD 0.01 = Critical Difference at 1% level of significance, CV = Coefficient of Variation. Means followed by a common letter are not significantly different at 1% level by Tukey’s (HSD)

### Increasing phenolic content in resistant maize genotypes

**Fig. 4:**
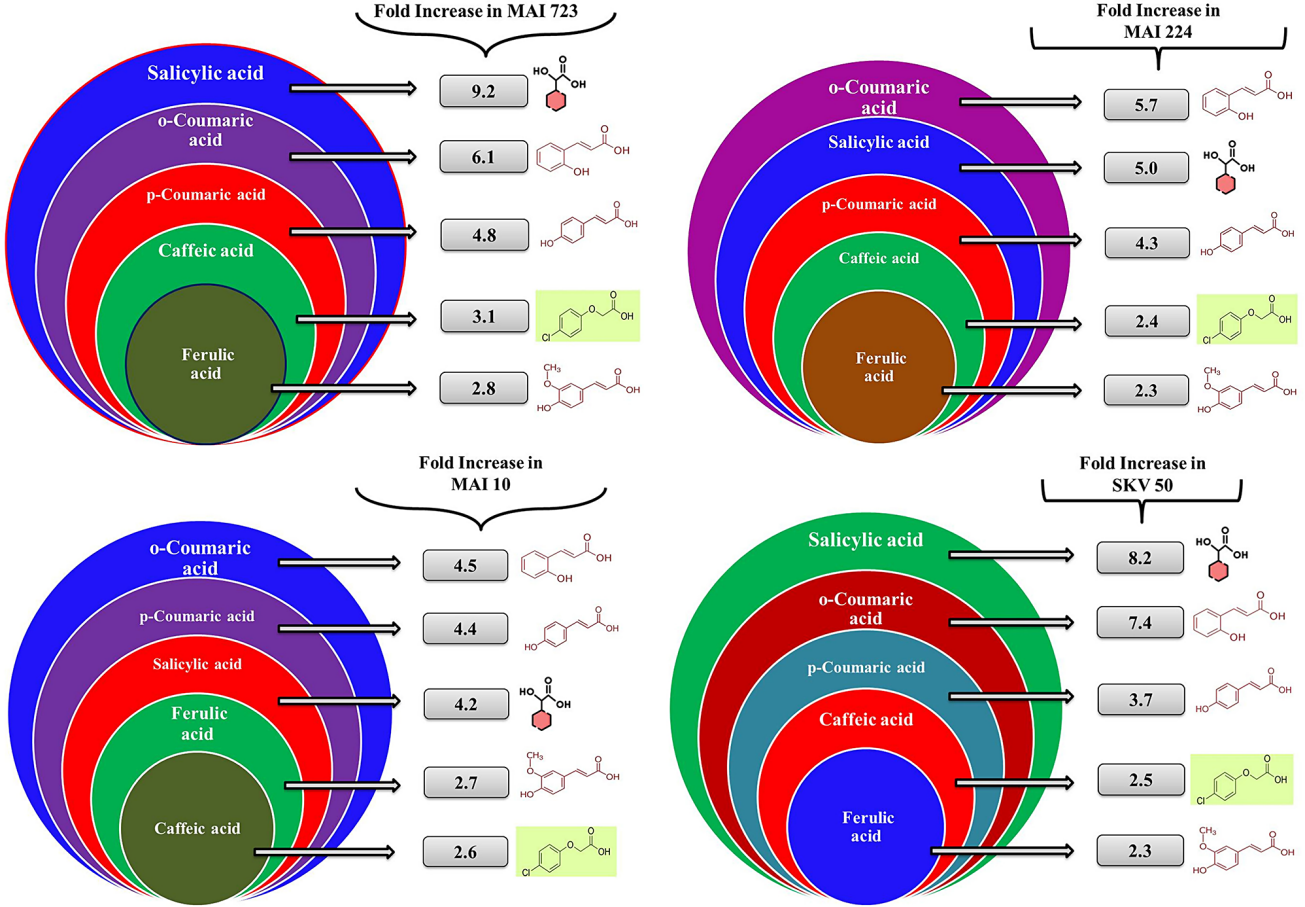
Enhanced Phenolic Content in Resistant Maize Genotypes

Resistant maize genotypes, including MAI 10, SKV 50, MAI 723, and MAI 224, exhibited a substantial elevation in phenolic acids—Salicylic, P-Coumaric, O-Coumaric, Caffeic, and Ferulic Acids—compared to the susceptible African tall genotype (Fig. 4). Salicylic acid displayed a 4.2 to 9.2-fold increase, P-Coumaric acid exhibited a 3.7 to 4.8-fold rise, O-Coumaric acid revealed a 4.5 to 7.4-fold increase, Caffeic acid demonstrated a 2.4 to 3.1-fold elevation, and Ferulic acid showed a 2.3 to 2.8-fold increase. These findings suggest potential associations with heightened resistance against *Peronosclerospora sorghi*, the causative agent of MDM disease, shedding light on the intricate interactions between plant phenolics and disease resistance mechanisms in maize. The significant increase in phenolic compounds observed exclusively across resistant maize genotypes further underscores their potential role in conferring resistance against *Peronosclerospora sorghi*.

### Flavonoid content and fold increase in resistant genotypesto maize downy mildew

The flavonoid analysis revealed significant variations in the concentrations of key compounds, particularly Naringenin, Catechin, and Epicatechin, among the tested maize genotypes, including African Tall, MAI 10, SKV 50, MAI 723, and MAI 224 (Table 4). Naringenin, known for its antioxidant properties, showed noticeable differences across the genotypes. While African Tall recorded 34.4 µg/g, the resistant genotypes showed higher concentrations: MAI 10 (88.3 µg/g), SKV 50 (81.1 µg/g), MAI 723 (81.2 µg/g), and MAI 224 (130 µg/g). This recommends a potential association between elevated Naringenin levels and resistance to MDM in the tested genotypes. Catechin, another crucial flavonoid with antioxidant and anti-inflammatory properties, showed substantial variability. African Tall had a concentration of 22.9 µg/g, whereas the MAI 10 (124.4 µg/g), SKV 50 (62.2 µg/g), MAI 723 (73.6 µg/g), and MAI 224 (117.9 µg/g) exhibited significantly higher levels. This underscores the potential role of Catechin in the defence mechanisms against MDM. Epicatechin, also displayed notable differences. African Tall indicated a concentration of 1.3 µg/g, while MAI 10 indicated the highest level at 8.2 µg/g, followed by SKV 50 (7.4 µg/g), MAI 723 (7.2 µg/g), and MAI 224 (7.7 µg/g). These findings propose a potential link between increased Naringenin, Catechin, and Epicatechin levels and resistance to MDM in the tested maize genotypes.

**Table 4:** Quantitative LCMS analysis of flavonoid content in Maize: resistant genotypes vs. susceptible African Tall

Flavonoids (µg/g)*	African Tall	MAI 10	SKV 50	MAI 723	MAI 224	SEM	CD 0.01	CV
Catechin	22.9 ^**e**^	124.4 ^**a**^	62.2 ^**d**^	73.6 ^**c**^	117.9 ^**b**^	0.07	0.28	0.14
Naringenin	34.4 ^**d**^	88.3 ^**b**^	81.1 ^**c**^	81.2 ^**c**^	130 ^**a**^	0.08	0.35	0.17
Epicatechin	1.3 ^**e**^	8.2 ^**a**^	7.4 ^**c**^	7.2 ^**d**^	7.7 ^**b**^	0.04	0.11	0.96
Rutin	2.2 ^**a**^	2.3 ^**a**^	0.7 ^**d**^	1.9 ^**b**^	1.1 ^**c**^	0.03	0.14	3.39
Myricetin	2.2 ^**a**^	1.4 ^**b**^	0.7 ^**d**^	1.1 ^**c**^	1 ^**c**^	0.03	0.15	4.73
Apigenin	1.3 ^**b**^	1.0 ^**c**^	0.6 ^**d**^	4.7 ^**a**^	0.7 ^**d**^	0.03	0.12	2.99
Epigallocatechin	2.5 ^**a**^	1.0 ^**c**^	2.1 ^**b**^	0.3 ^**e**^	0.9 ^**d**^	0.02	0.1	3.14
Luteolin	0.9 ^**b**^	0.9 ^**b**^	0.3 ^**c**^	1.3 ^**a**^	0.9 ^**b**^	0.02	0.07	3.53
Quercetin	0.5 ^**b**^	0.4 ^**c**^	0.3 ^**d**^	1.3 ^**a**^	0.4 ^**c**^	0.01	0.04	3.05
Fisetin	0.1 ^**a**^	0.1 ^**a**^	0.1 ^**a**^	0 ^**b**^	0.1 ^**a**^	0.01	0.02	11.08
Hesperetin	0 ^**b**^	0.1 ^**a**^	0 ^**b**^	0.1 ^**a**^	0 ^**b**^	0.01	0.03	17.75
Umbelliferone	0 ^**a**^	0 ^**a**^	0 ^**a**^	0 ^**a**^	0 ^**a**^	0	0	0
Galangin	0 ^**a**^	0 ^**a**^	0 ^**a**^	0 ^**a**^	0 ^**a**^	0	0	0
Kaemperol	0 ^**a**^	0 ^**a**^	0 ^**a**^	0 ^**a**^	0.1 ^**a**^	0	0	0
Eriodictyol	0 ^**a**^	0 ^**a**^	0 ^**a**^	0 ^**a**^	0 ^**a**^	0	0	0

* mean of four replications, SEM = Standard Error of the Mean, CD 0.01 = Critical Difference at 1% level of significance, CV = Coefficient of Variation. Means followed by a common letter are not significantly different at 1% level by Tukey’s (HSD)

**Fig. 5:**
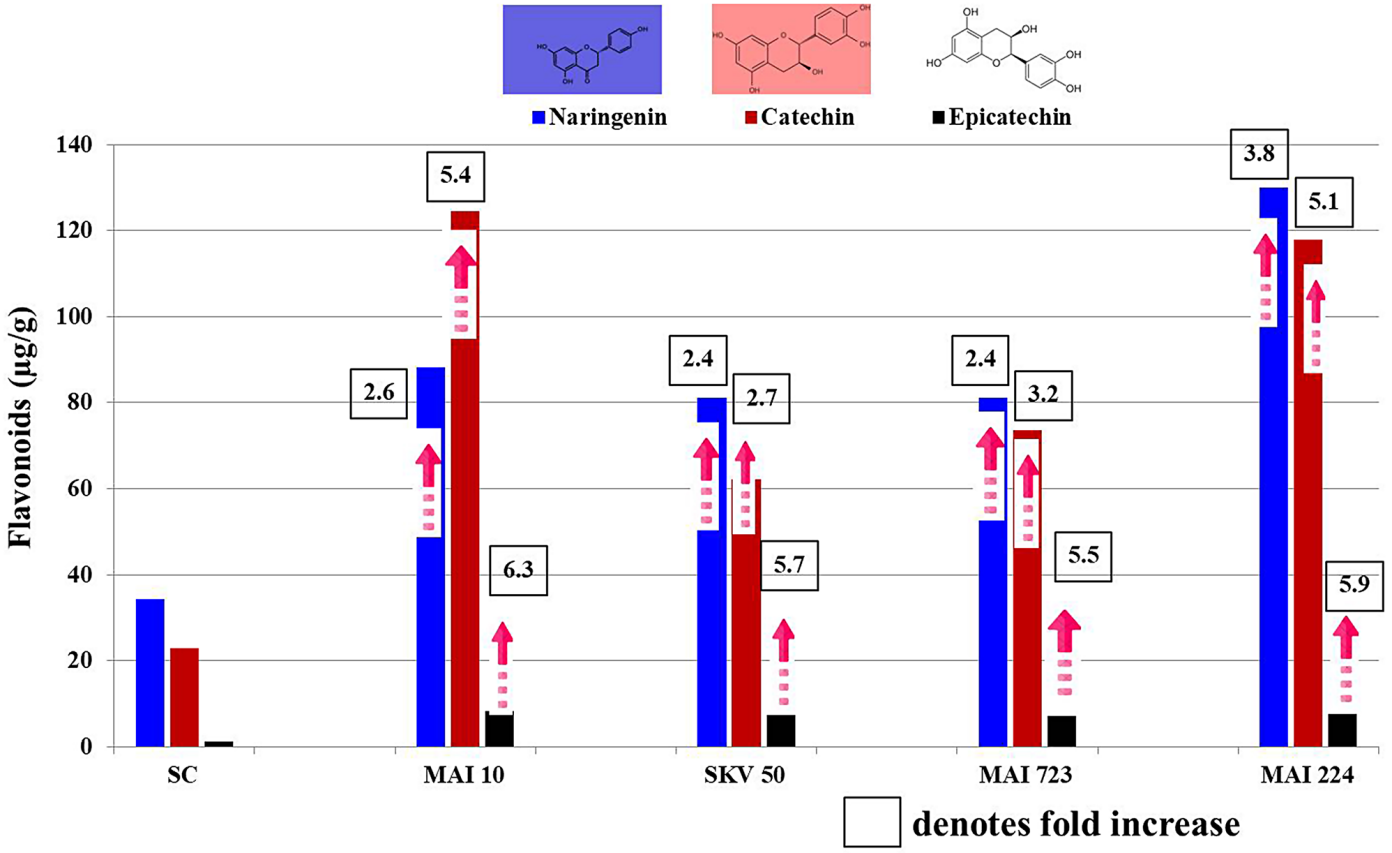
Elevated Flavonoid Levels in Resilient Maize Genotypes

The resistant maize genotypes (MAI 10, SKV 50, MAI 723, and MAI 224) displayed significant elevations in flavonoid content, particularly Naringenin, Catechin, and Epicatechin, compared to the susceptible African Tall genotype (Fig. 5). Naringenin exhibited a remarkable range of 2.4 to 3.8-fold increase, Catechin showed a range of 2.7 to 5.4-fold increase, and Epicatechin demonstrated enhancements with a range of 5.5 to 6.3-fold increase. This exclusive increase in flavonoid levels across resistant genotypes further underscores their potential role in the mechanisms of resistance against *Peronosclerospora sorghi*, the causative agent of MDM disease, in maize.

## Discussion

### Prevalence of MDM

The study investigated the intricate spatial and temporal dynamics of MDM incidence, revealing variations across districts and years that underscore the influence of localized factors. Environmental conditions, agronomic practices, and pathogen diversity were found to shape this disease landscape. This aligns with prior research [36], highlighting the interplay between virulent pathogens, susceptible hosts, and a conducive environment which play a pivotal role, impacting host physiology, resistance mechanisms, and pathogen growth. Climate change amplifies this impact, with variations in rainfall, humidity, temperature, and sunlight altering the host-pathogen equilibrium. Such changes, driven by climate shifts, can reshape disease prevalence, echoing the disease variations [37]. highlighted meteorological conditions, such as air currents that disperse pathogenic spores and contribute to regional epidemics, play a role [38]. Mentioned, the genetic diversity within downy mildew populations and the pathogen’s evolution contribute to spatial variability in disease incidence, as different regions may host distinct pathogen populations with varying virulence factors. Temporal variation can also be attributed to environmental conditions that change over time, such as weather patterns that affect the pathogen’s lifecycle and infection capability [39]. In summary, spatial and temporal variations in downy mildew disease are influenced by a complex interplay of factors including environmental conditions, pathogen dispersal mechanisms, genetic diversity of the pathogen, and the host’s resistance responses. These variations necessitate continuous monitoring and adaptation of disease management strategies to effectively control downy mildew in various crops [37–39]. The study underscores the spatial and temporal dynamics of MDM in the southern districts of Karnataka. These regions require vigilant attention to prevent potential epidemics. The findings carry substantial implications for disease management strategies, offering valuable insights into the evolving dynamics of MDM. They emphasize the need for adaptive strategies in response to changing climatic conditions and the shifting disease landscape.

### Fungicide interventions for MDM management

The control of maize downy mildew caused by *Peronosclerospora sorghi* primarily relies on the use of chemical fungicides like Metalaxyl [40]. However, the emergence of resistance to Metalaxyl by *Peronosclerospora sorghi* in Texas has raised worries about its long-term efficacy [41]. This underscores the need for alternative combination and new fungicide formulations for MDM control. The introduction of new-generation fungicides becomes imperative to sustain control of major pathogens in agriculture [42]. These fungicides, with new modes of action, play a vital role in resistance management strategies [43]. Their ecologically safer profile, lower dosage requirements, and site-specific action make them valuable tools in modern disease management [44].Fungicides with new modes of action have evolved significantly, with compounds such as strobilurins and triazole fungicides evolving as potent tools for disease management [45]. These new-generation fungicides offer innovative solutions for disease control, with new modes of action aligned with sustainable agricultural practices. The proactive use of these fungicides, guided by FRAC guidelines, remains essential to ensure their continued efficacy in disease management [43, 44].

Our finding is in supporting with other reports. The management of downy mildew across various crops has been shown to be successfully achieved through the use of fungicides, particularly combinations such as metalaxyl + mancozeb and azoxystrobin + difenoconazole. In the perspective of onion downy mildew, the combination of metalaxyl + mancozeb has been effective in reducing disease severity and increasing yield [46–49]. Similarly, for the management of downy mildews in other crops, such as boysenberry and basil, metalaxyl-M has been used in combination with other fungicides, though its efficacy varied with disease pressure [50, 51]. Azoxystrobin, often used in blend with other fungicides, has shown effective in certain conditions but showed resistance issues in others[50, 52, 51].Furthermore, although not expressly studied, the combination of azoxystrobin and difenoconazole would most likely benefit from azoxystrobin’s broad-spectrum activity and difenoconazole’s systemic characteristics, based on azoxystrobin’s overall efficacy in fungicide combinations. Therefore, the use of these fungicides should be considered as part of an integrated disease management plan, taking into account local conditions and pathogen sensitivity [53–55]. In conclusion, the present study underscores the pivotal role of fungicides in managing MDM. The integrated approach demonstrated impressive results in reducing disease incidence and enhancing crop yield.

### Phenolic and flavonoid mastery: unveiling maize plant’s defence against MDM

The biochemical defence mechanism may consist of the presence or absence of a specific chemical or collection of compounds in a host plant that inhibits the pathogen’s growth and reproduction. The biochemical pathway may exist prior to infection or be formed as a result of the host-pathogen interaction. The exploration of the dynamic interplay between phenolic compounds, encompassing both phenolic acids and flavonoids, pathogenic challenges, and resistant and susceptible lines in maize has yielded intriguing insights that significantly contribute to our understanding of plant defence mechanisms and potential strategies for disease management. The study offers a comprehensive overview of the alterations in phenolic compound levels following resistant and susceptible lines illuminating the intricate biochemical responses within the maize plant.

### Phenolic acids as defence molecules

The Phenolic acids play a crucial role as defence molecules in plants, particularly in response to pathogens like *Peronosclerospora sorghi*. Comparative analysis of susceptible and resistant genotypes revealed a notable increase in phenolic acid levels in response to the pathogen. Among the 18 phenolic acids studied, five stood out for their distinct response patterns. Salicylic acid, a key signalling molecule in plant defence pathways, consistently increased in resistant genotypes, indicating effective defence activation. p-Coumaric acid and o-Coumaric acid, known for their antioxidant and pathogen defence properties respectively, also showed significant increases in resistant genotypes, suggesting robust defence mechanisms. Caffeic acid displayed varied responses, reflecting the complex balance between defence activation and metabolic pathway modulation in resistant genotypes. Ferulic acid, important for plant cell wall strength, increased significantly in resistant genotypes, indicating enhanced structural fortification and potential influence on defence signalling. This implies that resistant genotypes prompt the production of diverse phenolic acids, potentially enhancing both structural fortification and defence signalling pathways.

### Flavonoids as defence molecules

Flavonoids play a pivotal role in plant defence against pathogenic attacks. The observed variations in flavonoid content in response to susceptible and resistant genotypes highlight the plant’s dynamic biochemical response to challenges. Among the 15 compounds analyzed, Naringenin, catechin, and epicatechin exhibit overexpression in the resistant genotypes, while the remaining compounds are present in relatively smaller amounts. Naringenin, catechin and epicatechin are recognized for their antimicrobial properties, demonstrated significant increases in content following the infection with *Peronosclerospora sorghi*. This suggests that the resistant genotypes may trigger the synthesis of specific flavonoids as part of the plant’s defence strategy, enhancing its resistance against pathogenic invaders. The distinctive responses of Naringenin, catechin and epicatechin to the experimental conditions underscore the complexity of flavonoid biosynthesis regulation. The fold increase analysis indicated that the resistant genotypes consistently amplified the production of these flavonoids. This differential response suggests a temporal aspect to the activation of flavonoid biosynthesis pathways in the plant’s defence against pathogenic challenges in the resistant genotypes.

This study sheds light on the interplay between phenolic acids, flavonoids, and pathogenic challenges in both susceptible and resistant maize genotypes, suggesting promising avenues for further research. Understanding the molecular mechanisms behind phenolic acid and flavonoid biosynthesis could deepen our understanding of plant defence mechanisms. Discovering the interactions among these compounds may reveal synergistic effects enhancing overall resistance. In summary, analysing phenolic acid and flavonoid levels in resistant maize genotypes provides valuable insights into plant defence mechanisms, bridging the gap between research and agricultural practices for more effective disease management and sustainable crop production.

The findings of this study align with previous research that highlights the intricate defence responses plants activate in the face of microbial attacks. The key role of phenolics and flavonoids in induced defence against downy mildew is buoyed by several studies that prove their accumulation in reply to pathogen infection. Phenolic compounds are connected with the reinforcement of cell walls, thereby contributing to the physical barrier against pathogen entry [56–58]. The activation of phenylalanine ammonialyase (PAL), aimportant enzyme in the phenylpropanoid pathway leading to the production of phenolics, is observed in resistant cultivars of pearl millet upon infection with *Sclerospora graminicola* This proposes a positive correlation amid PAL activity, phenolic accumulation, and the degree of resistance to downy mildew [56].The instrumentation of induced resistance includes a complex network of signal transduction pathways, in which phenolic acids emerge as essential signalling molecules [59, 60]. Within this network, certain phenolic acids exhibit robust antifungal properties [61, 62]. The accumulation of phenolic compounds at the site of challenge also serves to fortify the cell wall, an effect augmented by the localized generation of reactive oxygen species. This phenomenon drives activities such as cell wall cross-linking, antimicrobial action, and the initiation of defence signalling [63].

Catechin and epicatechin, in particular, have been recognized as compounds that contribute to the defence of plants against various biotic stresses. In the context of induced defence against plant pathogens, catechin has been shown to participate in the defence of tea plants against herbivores by accumulating in response to jasmonic acid, ethylene, and auxin mediated signalling pathways [64]. Similarly, epicatechin is produced in tea plants through the activity of anthocyanidin reductase, which is part of the plants defence against pathogens [65].Interestingly, while catechin and epicatechin have been implicated in defence responses, naringenin was not oxidized by the DPPH radical nor by air oxygen, suggesting a different role or a lesser involvement in direct antioxidant defence mechanisms compared to catechin [66]. This could indicate a more nuanced role for naringenin in plant defence, potentially involving signalling or other indirect mechanisms. In summary, catechin and epicatechin are involved in the induced defence against plant diseases, as evidenced by their accumulation in response to signalling molecules associated with plant defence pathways [64, 65]. Naringenin, on the other hand, may play a different role in plant defence, as it does not exhibit the same direct antioxidant properties as catechin [66]. Further research could elucidate the specific functions of these flavonoids in plant defence mechanisms.

The findings of this study not only deepen our understanding of the biochemical mechanisms underlying maize resistance to downy mildew but also highlight several avenues for future research and practical applications. Firstly, exploring the genetic basis of phenolic and flavonoid biosynthesis through advanced genomic techniques could provide further insights into the molecular pathways driving resistance. Integrating these insights with genomic selection and genetic engineering approaches may lead to the development of maize varieties with enhanced, stable resistance. Additionally, practical applications of our findings could involve the implementation of marker-assisted selection (MAS) in breeding programs to accelerate the development of MDM-resistant cultivars. Field trials across diverse agro-ecological conditions will be essential to validate the effectiveness and stability of these resistance traits. Furthermore, the application of the identified biochemical markers in precision agriculture could aid in more targeted management strategies, optimizing fungicide use and improving overall crop resilience. Ultimately, bridging the gap between biochemical research and practical breeding applications holds the potential to significantly advance sustainable maize production and mitigate the impact of MDM on food security.

## Conclusion

This comprehensive study sheds light on key aspects of MDM management, including disease prevalence, fungicide efficacy, host plant resilience, and biochemical defence mechanisms in maize plants. Across the southern districts of Karnataka, India, our research reveals fluctuating disease incidences across different rainy seasons. Through rigorous experimentation, we identified a highly effective strategy for managing MDM, involving a combination of seed dressing and targeted foliar spray. This approach led to a significant reduction in disease incidence and substantially enhanced maize yield. Furthermore, our investigation into maize inbred lines uncovered genotypes that consistently displayed stable resistance to MDM over multiple seasons, providing promising genetic resources for future breeding efforts. Analysis of biochemical dynamics revealed significant increases in essential phenolic and flavonoid compounds in resistant genotypes. These findings underscore the importance of chemical defence responsiveness in maize plants and offer valuable insights into effective disease mitigation strategies and grain yield enhancement. By integrating crucial aspects of crop security, this research contributes to the revitalization of maize downy mildew management and fosters resilience against MDM challenges.

## Electronic supplementary material

Below is the link to the electronic supplementary material.

Supplementary Material 1

Supplementary Material 2

Supplementary Material 3

Supplementary Material 4

Supplementary Material 5

## Data Availability

All data supporting the findings of this study are included within the manuscript and supplementary materials. This includes Tables 1, 2, 3 and 4, Supplementary Tables 1–4, Figs. 1, 2, 3a and b and 4, and 5, as well as Supplementary Fig. 1.

## Abbreviations

MDM: maize downy mildew
LCMS/MS: Liquid Chromatography-Mass Spectrometry
PDI: Per cent Disease Incidence
PROC: Per cent Reduction over Control
PIOC: Per cent Increase over Control
BC ratio: Benefit and Cost Ratio
RC: resistant Check
SC: Susceptible Check
R: Resistant
MR: Moderately Resistant
MS: Moderately Susceptible
S: Susceptible
SD: Seed Dressing
FS: Foliar Spray
WP: Wettable Powder
WS: Water-Soluble Powder
SC: Suspension Concentrate
mL: Milliliter
N: Normality (or Normal solution)
NaOH: Sodium Hydroxide
HCL: Hydrochloric Acid
MS-Grade: Mass Spectrometry-Grade
