# Supplementary figures and images for: Revitalizing maize downy mildew management: harnessing new-generation fungicides and host plant resistance
Source: BMC Plant Biol. 2025 Feb 17;25:211. doi: 10.1186/s12870-024-05882-z (PMC11831823; doi:10.1186/s12870-024-05882-z)

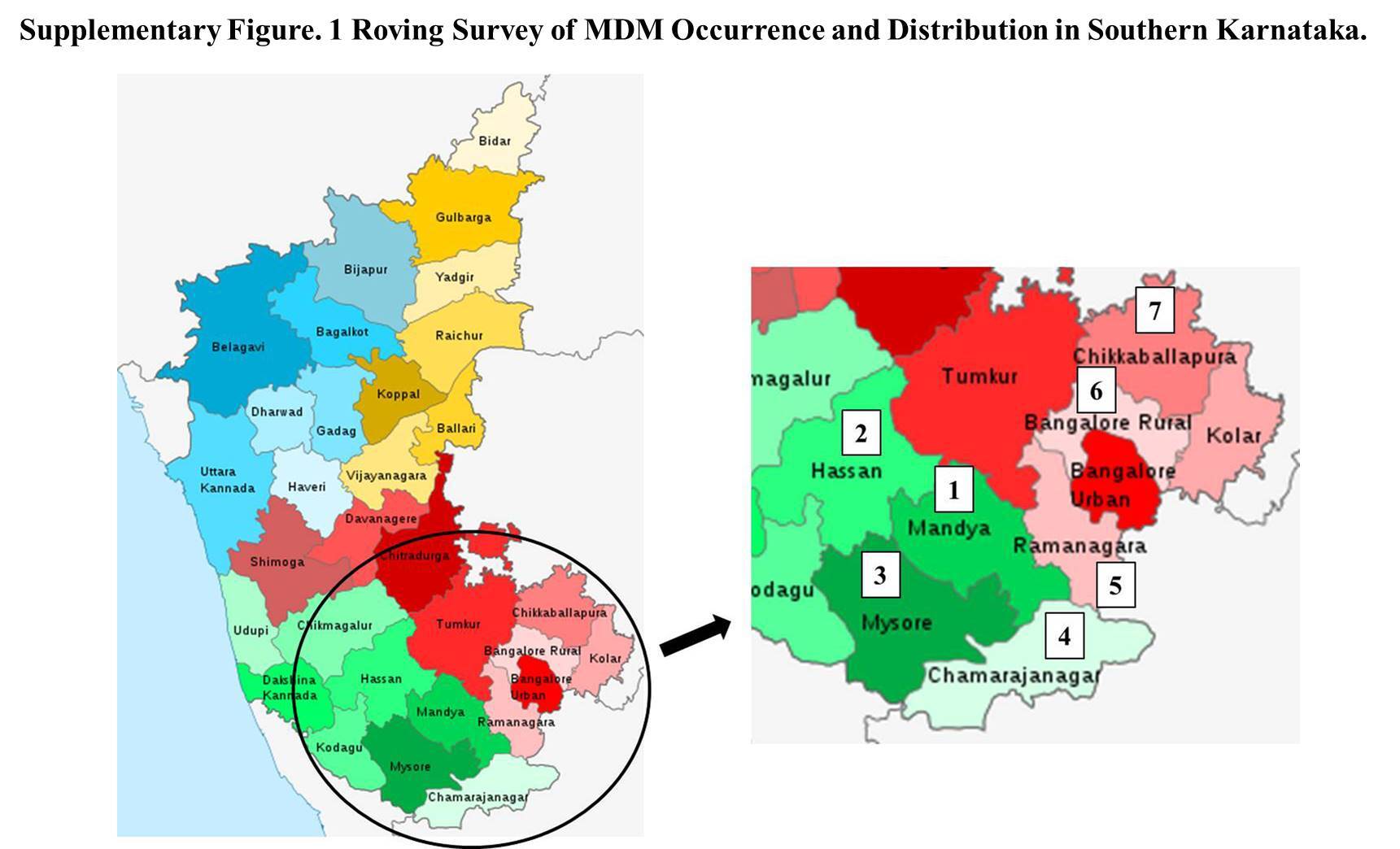

Supplement: Supplementary file 4 — Supplementary Material 4 [file 12870_2024_5882_MOESM4_ESM.jpg]
